# How Can We Measure Alcohol Outlet Density Around Schools? A Comparison Between Two Buffer-Based Methods

**DOI:** 10.1007/s11524-023-00740-z

**Published:** 2023-06-23

**Authors:** Irene Martín-Turrero, Xisca Sureda, Francisco Escobar, Usama Bilal, Maitane Berasaluce, Roberto Valiente

**Affiliations:** 1 Public Health and Epidemiology Research Group, School of Medicine, University of Alcalá, Campus Universitario - Crta. de Madrid-Barcelona, Km. 33,600. Alcalá de Henares, 28871 Madrid, Spain; 2 Department of Epidemiology & Biostatistics, Graduate School of Public Health & Health Policy, City University of New York, New York, NY 10027 USA; 3 Tobacco Control Research Group, Institut d’Investigació Biomèdica de Bellvitge-IDIBELL, l’Hospitalet de Llobregat, Barcelona, Spain; 4 Centro de Investigación Biomédica en Red de Enfermedades Respiratorias (CIBERES), Madrid, Spain; 5 Department of Geology, Geography and Environmental Sciences, University of Alcalá, Alcalá de Henares, Madrid, Spain; 6 Urban Health Collaborative, Drexel Dornsife School of Public Health, Philadelphia, PA 19104 USA; 7 Department of Epidemiology and Biostatistics, Drexel Dornsife School of Public Health, Philadelphia, PA 19104 USA; 8 Center for Research on Environment, Society and Health (CRESH), School of GeoSciences, University of Edinburgh, Edinburgh, UK; 9 SPECTRUM Consortium, London, UK

**Keywords:** Alcohol accessibility, Schools, GIS, Buffers, Bland-Altman analysis

## Abstract

Measuring the density of alcohol outlets around schools is a critical step towards understanding the drivers of drinking among adolescents. Different methodologies have been used in the literature for this purpose, but the implications of using one methodology or another have not been clearly assessed. Our aim was to compare different methods to measure alcohol outlet density and highlight under which characteristics of the environment might be best using each approach. We used Geographic Information Systems to geolocate schools (*n* = 576) and alcohol outlets (*n* = 21,732) in Madrid. We defined the density of alcohol outlets as the number of establishments within an area of 400 m around schools measured using two buffering methods: crow flies’ and street network distances. We evaluated the agreement between both methods visually and through regression models, including street connectivity, population density, and density of recreational venues as predictors of disagreement. The density of alcohol outlets around schools was higher using crow flies’ distances compared to street network distances. The differences between methodologies were wider in areas of higher density of outlets, especially in the downtown areas, where there are higher population density and street connectivity. Our results suggest that the spatial characteristics and morphology of the study area (e.g., street connectivity and population density) should be considered when deciding the methodology to be used to measure alcohol outlet density. Future studies should explore the implications of different exposure measures in their association with drinking prevalence and consumption patterns among different geographical contexts.

**Supplementary Information:**

The online version contains supplementary material available at 10.1007/s11524-023-00740-z.

## Introduction

Sociodemographic, geographic, and cultural factors influence alcohol consumption [1, 2]. Among these factors are the physical, social, and cultural features of the environment. Physical features include the density of or proximity to alcohol outlets in the neighborhoods where people live, work, and play. For example, higher levels of alcohol outlets in the environment reduce the time and effort that people need to obtain alcohol products and are associated with higher rates of drinking and health and social problems [3, 4]. This might be especially problematic in areas near schools with a high presence of vulnerable populations, such as adolescents [5, 6]. Social features of neighborhoods include socio-economic deprivation, which is positively associated with a higher density of alcohol outlets [6, 7].

However, there are still inconsistent results in the literature regarding the influence of the urban context in individual behaviors [8, 9]. A wide range of methods has been used to evaluate this exposure, measuring the accessibility to and availability of alcohol around spaces frequented by individuals (i.e., residences, schools, or working places) [10]. Such heterogeneity in the methods might be the cause of the inconsistencies found in the results in the literature. For instance, studies using alcohol outlet density measures to examine the contextual drivers of alcohol consumption have used a diversity of calculations: the number of alcohol outlets per 1000 inhabitants by the district of residence [11], the number of outlets within 1 km Euclidean buffers from the participant’s home [12], or the density of outlets per square kilometer within the participant’s census tract of residence [13], among others. The proliferation of these diverse methods has occurred in consonance with the rapid technological developments in Geographical Information Systems (GIS) during the last years.

In addition to the methods commented above, there is an increasing number of studies that use Global Positioning System (GPS) technologies to generate exposure measures at an individual level, considering the exact areas they usually visit [8]. These approaches constitute an applied example of the *Uncertain Geographic Context Problem* (UGCoP), developed by Chen and Kwan [14]. In brief, the UGCoP refers to the problem of how the effects of area-based attributes (e.g., the density of alcohol outlets) on individual behaviors (alcohol use) could be affected by how the neighborhood or study area in which we aim to measure the alcohol exposure is defined. However, the use of these complex GPS-based methods is highly expensive, time-consuming, and often difficult to interpret and translate into concrete policy actions. A common alternative to this method is the creation of buffers using GIS to approach the immediate (and hypothetical) neighborhoods of the residential or school area in which the individuals might satisfy their primary needs. A buffer is an area resulting from a distance calculation around a specific location (e.g., home or school coordinates), considering a given parameter of distance measured from that point [15].

Many studies in the literature have used buffers around the place of residence to evaluate the availability and accessibility that individuals have to different drugs [16, 17]. There are several forms to estimate buffers, being the most common ones those calculated with crow flies’ and street network distances. In brief, the crow flies’ buffers represent a circular area around a specific point, where the buffer radius is delineated using a straight line without considering obstacles in space. On the other hand, street network buffers are delineated using walkable distances through the streets, and thus, their shape is usually irregular. However, the complexity of the calculation and the accuracy of these options make this choice not trivial. From a methodological point of view, street network buffers show a more realistic view of alcohol availability and accessibility by considering only the paths through which an individual would walk to reach a store or alcohol outlet. In turn, crow-fly buffers do not account for physical barriers to the distance accessibility to alcohol outlets, such as the presence of water bodies, railway tracks, non-pedestrian paths, and crosswalks. However, the use of crow flies’ might provide some advantages towards street network ones in some cases, as well. Crow-fly buffers require less complex computation and less software requirements to its calculation. In addition, data on the street network are not always available and may contain important inaccuracies depending on the study area (e.g., cities in developing countries may have a poor spatial data infrastructure).

The specific implications of using either crow flies’ or street network-based buffers in the results obtained from the exposure measures have not been assessed in the literature [16, 18]. Moreover, we hypothesize that these implications may vary according to the characteristics of the study area, in terms of their geography or urban morphology. To better understand the implications of this choice, we aimed to calculate the density of alcohol outlets around secondary schools in the city of Madrid using these two types of buffers. The objective is to compare both calculations and to point out the advantages of each one, in relation to the neighborhood geographic and urban morphology characteristics.

## Methods

### Study Area

This study was conducted in the city of Madrid, which constitutes the largest municipality in Spain with more than 3.3 million inhabitants, concentrated in a spatial extension of 606 sq. km [19], which translates into an average population density of 5445.54 inhabitants per sq. km. Madrid is administratively divided into 2443 census tracts, which are the smallest official spatial statistical unit in Spain and enclose an area with an average population of 1500 inhabitants.

Census tracts located in Madrid’s city center are characterized by a high population density, a dense and irregular street network, and a high concentration of recreational venues, in comparison to those census tracts located in the outskirts in which the street network pattern leads to a lower population density, with a predominantly residential land use. For instance, when comparing Madrid’s downtown (e.g., Centro District) and a typical periphery neighborhood (e.g., Barajas District), central areas present a mean population density which is 22 times higher (25,233.92 vs 1118.15 inhabitants per sq. km, respectively), a recreational venue density 11 times bigger (344.83 vs 30.34 recreational venues per sq. km.; mean whole city = 58.52), and a mean street connectivity density 3 times greater (1474.57 vs 500.14 intersections per sq. km.; mean whole city value = 874.66) (data calculated by the authors).

### Study Design and Databases

We organized this study in three steps. First, we calculated the alcohol outlet density around secondary schools (see the “*Alcohol outlet exposure measures*” section) using the two different buffer methods mentioned before. Second, we generated a set of geographic covariates using GIS, defined as population density, recreational venues density, and street network connectivity (see the “*Generation and description of geographic covariates*” section). We modeled these covariates as predictors of the disagreement between alcohol outlet densities around schools estimated using both crow flies’ and street network buffers. The population density covariate was defined to procure a proxy of the degree of urban compactness. Recreational venue density was used to introduce an indicator of land use diversity across the city. Specifically, areas with low recreational venue density were accounted as predominantly residential neighborhoods, while those with high recreational venue density were considered to have a mixed land use (i.e., more diversity: residential, commercial, business, etc.). Similarly, we included street network connectivity as the street network buffer calculation directly relies on the urban connectivity features in contrast to the crow flies’ buffer one. Finally, we performed a statistical comparison of factors driving differences between buffers (see the “*Comparison analysis between measures*” section).

### Alcohol Outlet Exposure Measures

Alcohol outlet density around each school was estimated using GIS. We collected data of the location of all secondary schools (*n* = 576; 129 public and 447 private), obtained from the open databases of the City Council in 2017. Each school in the database contained their respective geographic coordinates, which were used to create a GIS layer containing all schools as points. All GIS analyses were done with ArcGIS 10.6. (ESRI, Redlands, CA, USA).

We also obtained geolocated data of all on-premises (i.e., restaurants, bars, clubs, taverns, show bars, and wine cellars) and off-premises (i.e., supermarkets, small grocery stores, minimarkets, convenience stores, and liquor stores) alcohol outlets from the “*Census of premises and their activities, and restaurant terraces”* of the City Council of Madrid [20]. We downloaded information on the entire street network within the city of Madrid (updated to 2017) from the Open Street Maps databases (http://www.openstreetmap.org). The geographical topology of each street feature was checked to ensure that all intersections with valid turns between street segments were recognized in the street network calculation.

**Fig. 1:**
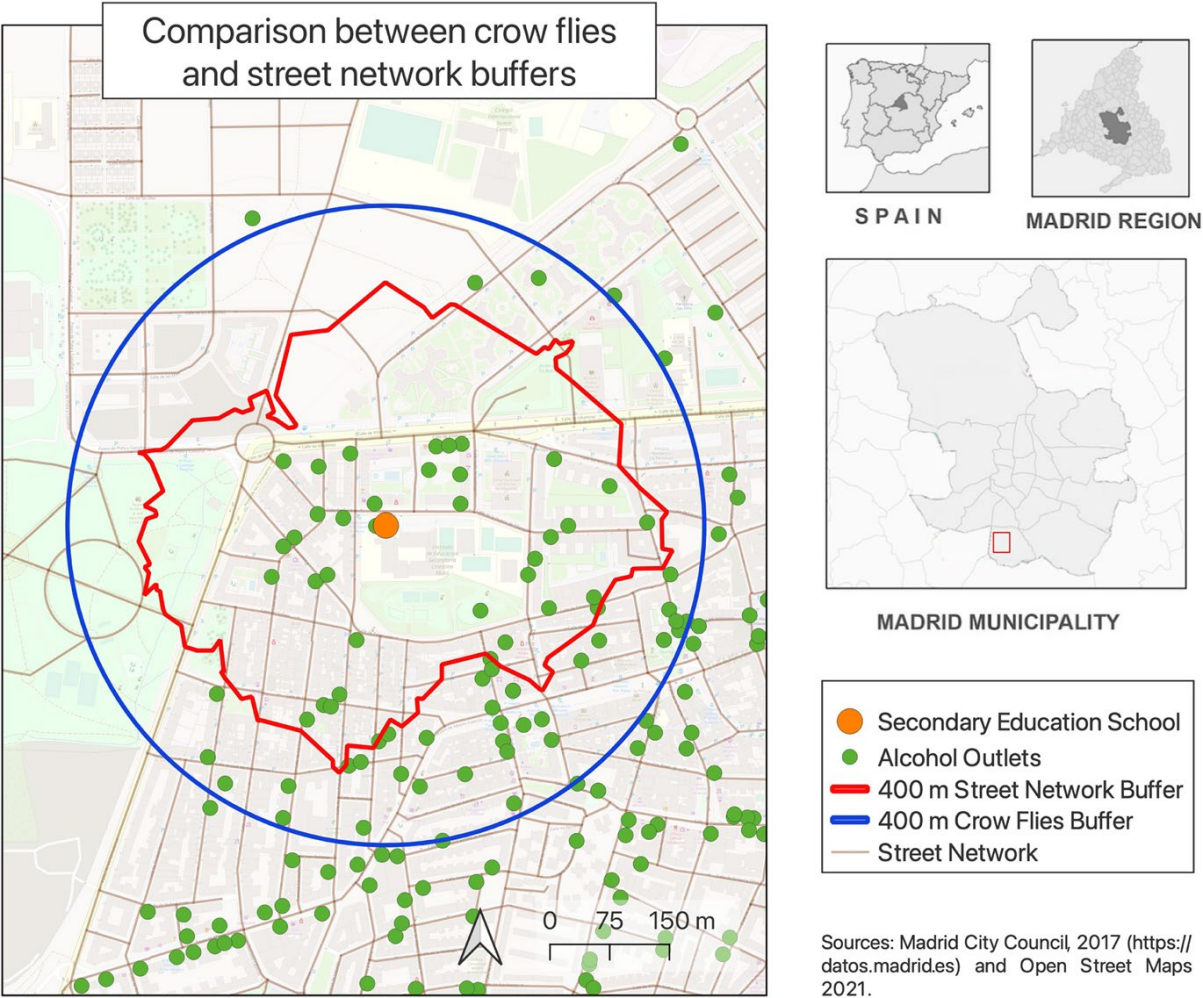
Differences between the areas covered by crow flies’ and street network buffers of the same size (400 m)

To calculate the density of alcohol outlets in 400 m crow flies’ and street network buffers, we counted the number of alcohol outlets within them. Sensitivity analyses were performed using 200 and 800-m buffers. These buffer sizes were chosen to allow analytic comparisons with previous studies on alcohol availability in Madrid [21]. Figure 1 illustrates the operationalization of both types of buffers in a school in the south of Madrid.

### Generation and Description of Geographic Covariates

To explore to what extent the potential differences observed in alcohol outlet density values might be explained by contextual factors [22], we measured population density, recreational venues density, and street network connectivity. We calculated population density (inhabitants per unit of sq. km) throughout the city of Madrid at the census tract level. Data of population at the census tract level was obtained from the Madrid Municipal Population Census (https://datos.madrid.es/) for 2017.

For recreational venue density, we obtained information about the total amount of recreational venues in the city from the “*Census of premises and their activities, and restaurant terraces”* of the City Council of Madrid [20]. Recreational venues were considered as places for leisure activities, including hospitality venues (bars, restaurants, cafés, pubs, and discos), tourist accommodations (hotels, hostels, and guesthouses), and other recreational and cultural centers such as theatres, cinemas, or museums. Most of these venues might be also categorized as on-premises alcohol outlets. A similar definition of recreational venues was used in previous studies [22]. We geocoded all of them and performed a point-density analysis using Kernel density estimations (KDE). The KDE are a geographic measure which provides a continuous density surface (i.e., raster) across the study area. In a continuous surface, the density values are depicted by pixels, which represent a specific portion of space within the study area. For our analysis, we estimated a 10 × 10 meters pixel size and a 1000 meters search radius. Then, we overlapped census tract areas over the KDE surface, and we extracted the mean density value of all the pixels within each census tract area to depict recreational venue density values at the census tract level.

For street connectivity, we identified each pedestrian street intersection within the study area running a topological analysis in GIS from the geocoded street network layer. We created a separate GIS point layer with the location of each intersection. A point density analysis using KDE was estimated using the same parameters as stated above for the recreational venue density calculation (10 meters pixels and 1000 meters search radius).

### Comparison Analysis Between Measures

To visually show the agreement between the value of the density of alcohol outlets measured by crow flies’ and street network buffers, we used the Bland-Altman plot. The Bland-Altman plot is a graphical method used to assess whether one method can replace the other with sufficient precision. In the *x*-axis, the mean of paired measurements is represented, and in the *y*-axis, the absolute difference between them. The graph includes a horizontal line that represents the mean difference between the measurements made with the two methods and two lines indicating distances of 1.96 and −1.96 standard deviations (SD), respectively. If the differences between the paired measurements follow approximately a normal distribution and the values tend to be stable throughout the measurement range, it is expected that 95% of those differences will fall within the limits of agreement [23, 24]. We also calculated the relative difference between densities by doing a density ratio (log-transforming both densities) for sensitivity analyses.

To measure the magnitude of the differences between the two methods taking the 400 m buffers, we fitted a negative binomial generalized linear model (NBGLM) where the crow flies’ measure was the dependent variable and the street network measure was the independent variable. We also included in the model the geographical covariates described above, plus an additional measure on neighborhood deprivation (socioeconomic status). We considered it appropriate to include this measure to account for variations due to potential social features of the environment in our analyses. Specifically, socioeconomic deprivation measures (SES) have been described as a good predictor for the density of alcohol outlets around schools too [7, 21]. We estimated the neighborhood deprivation measure by combining different socioeconomic indicators based on educational level, employment, occupational status, and housing prices. The composition of this measure has been previously described and used elsewhere [25].

All statistical analyses were performed using RStudio 1.4.1103 (R Core Team, 2019).

## Results

**Fig. 2:**
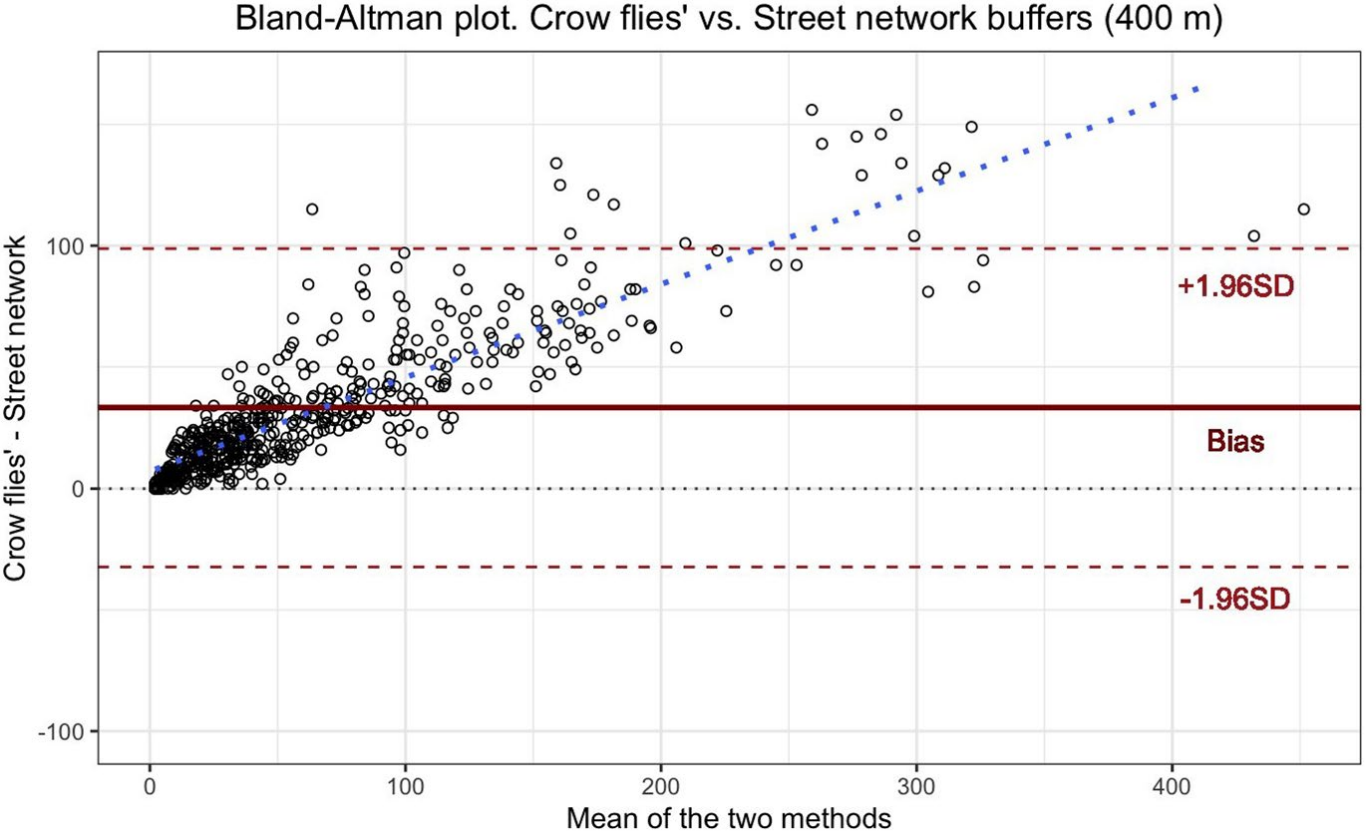
Bland-Altman plot compares the number of alcohol outlets around schools measured using 400 m crow flies’ buffers and street network buffers. The continuous line in the middle shows the average difference between the two methods, the dashed lines above and below (−1.96SD and 1.96 SD) are the limits of agreement between the two methods, and the dashed diagonal line represents the trend for the mean between methods and the differences between them

There were 576 secondary schools and 21,732 alcohol outlets in the city of Madrid. All of them were successfully geocoded, and the densities of alcohol outlets around schools were calculated. A representation of the Bland-Altman plot for the absolute comparison of the two density measurements is shown in Fig. 2, with the dashed line representing the line of equality (where both methods completely agree). Points above this line represent a higher density using the crow flies’ method, while points below this line represent a higher density using the street network method. On average, the crow-fly buffers measure 31.08 alcohol outlets more around schools than the street network buffers (limits of agreement: −33.99 to 96.14 alcohol outlets). The analyses assessing relative differences between the two methods showed no variation according to the basal density (i.e., 12/10 is the same as 120/100; however, 12–10 is smaller than 120–100). In other words, the two methods (crow flies’ and street network) give a relatively equal response regardless of basal density, but the absolute difference is greater with higher basal density. We observed the same trends when we analyzed the 200 m and 800 m buffers (see Supplemental Figures 1 and 2).

Our regression analysis showed that differences between the densities calculated by the two assessed methods increase a 1.0% (CI95: 0.8–1.1%) as the alcohol outlet values increase by one (i.e., for each 1 unit of increase in the count of outlets within street network buffers, the value of the density count within crow flies’ increases 1%) (Table 1). Higher population density and higher street connectivity were also related to greater differences between methods (1.0% increase per unit of change, respectively). We excluded recreational venue density and SES from the analyses since they did not contribute with significant information to the model.

**Table 1:** Association between the density of alcohol outlets measured by 400 m crow flies’ buffers (dependent variable) and 400 m street network buffers (independent variable). Adjusted by population density and street connectivity in Madrid, 2017

	Ratio of increase by 1 unit of change in alcohol outlet density within a buffer of 400 m IRR [95% CI]
Alcohol outlet density calculated using street network buffers	1.00961 (1.00825–1.01100)*
Population density (population/sq km)	1.00002 (1.00001–1.00002)*
Street connectivity	1.00105 (1.00085–1.00126)*

(*)*p* < 0.001

**Fig. 3:**
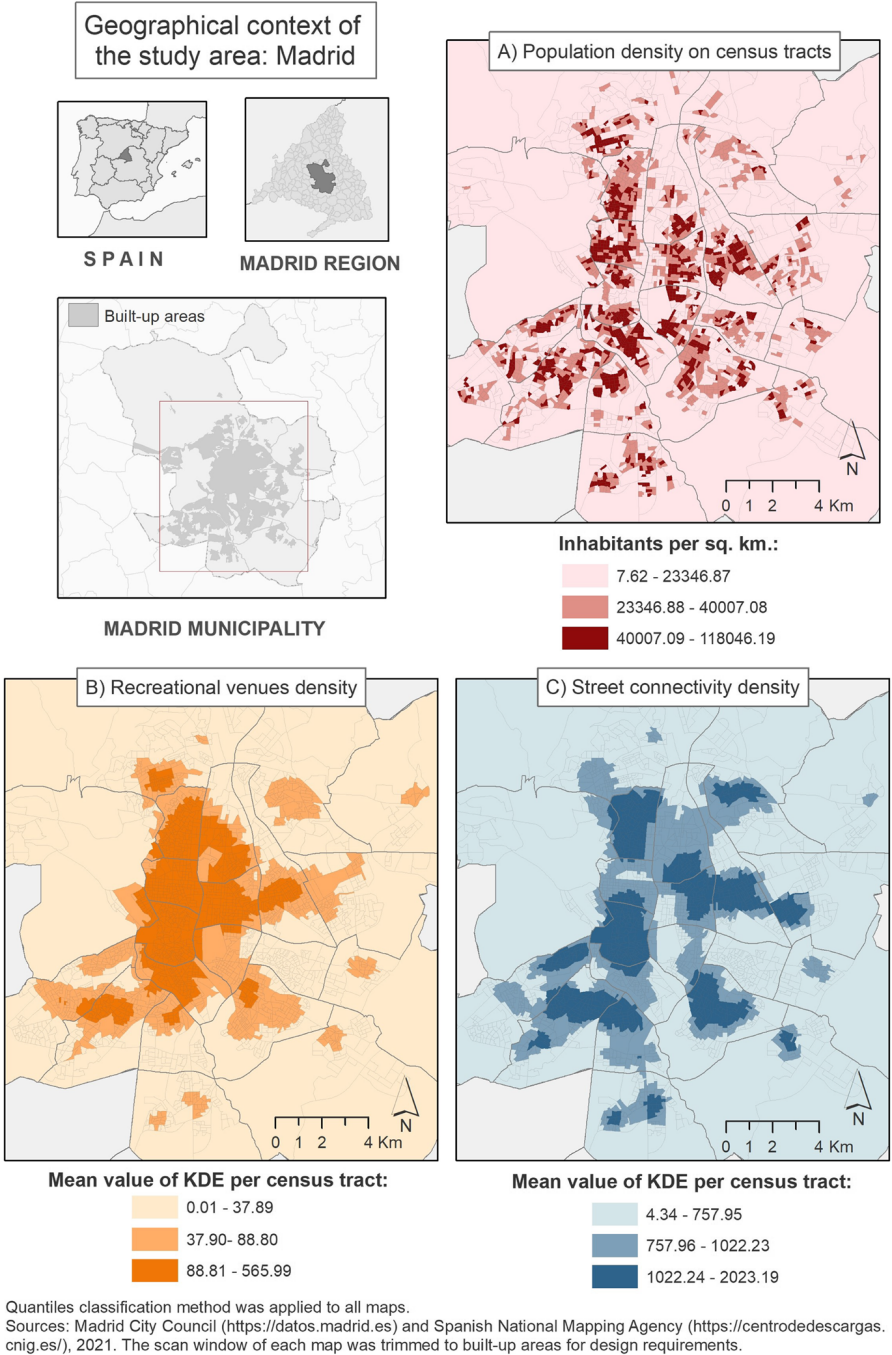
Geographical context of the study area: analyzing the spatial distribution of population density, recreational venues, and street connectivity

**Fig. 4:**
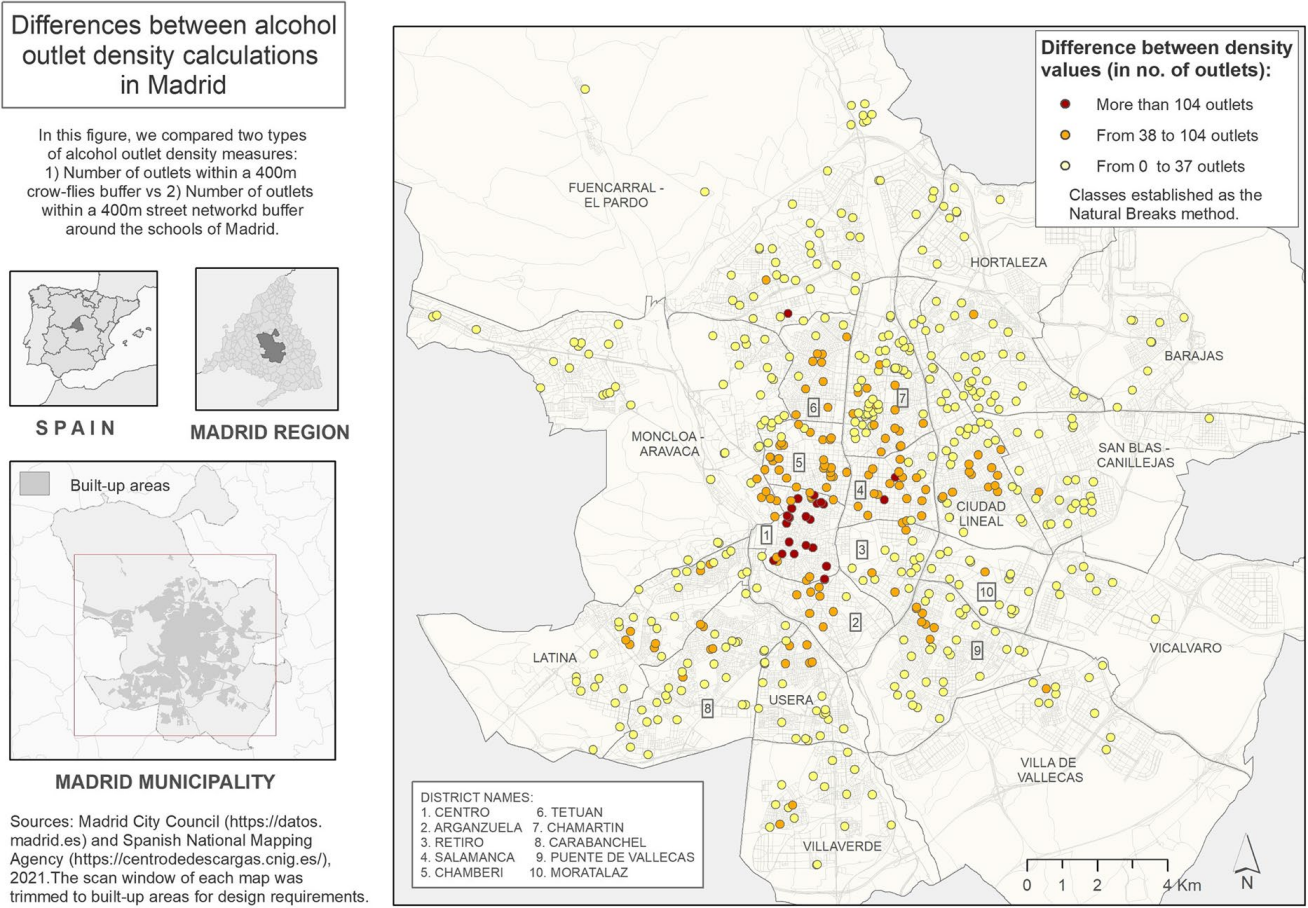
Spatial distribution of schools in Madrid, showing the difference between the density values of alcohol outlets measured using two different methods (400 m crow flies’ and street network buffers)

Figure 3 shows three maps characterizing the geographical context of the study area. On the top-right map, we observe the spatial distribution of population density, while both maps at the bottom represent the spatial distribution of the recreational venues (bottom-left) and the street connectivity density (bottom-right). In addition, Fig. 4 depicts the spatial distribution of the difference of density values obtained with the two studied methods (difference of count density within crow-fly buffers vs street network buffers). Figures 3 and 4 can be interpreted together and help us to visualize results from a spatial perspective and support the findings shown in Table 1. In Fig. 4, we observe that the schools surrounded by the highest alcohol density values are mainly located in the downtown area (Centro district). The downtown area also presents the highest values of recreational venues and street connectivity density, as shown in Fig. 3. Moreover, high population density values are also found in certain census tracts located in central areas. In other inner-city areas (e.g., Chamberi and Salamanca districts), we observe a visual correlation between high differences between both alcohol outlet density calculations and large densities of population, recreational venues, and street connectivity, as well.

## Discussion

This study proves that there are significant differences in the measurement of the density of alcohol outlets around schools when using crow flies’ compared to street network buffers. Density calculations based on crow flies’ buffers registered higher densities of alcohol outlets than those based on street network buffers of the same size, which varied according to other contextual factors of the study area, including population density and street network connectivity. This offers an interesting lecture, where we can interpret that the alcohol outlet density values per area remain stable (or even increase) as we move further from schools. Differences between measurements were larger in areas with higher population density and with a more densely connected street network. It is known that a crow flies’ buffer encompasses a larger territory (i.e., spatial extent) than a street network buffer of the same diameter; thus, it might also have a larger number of outlets inside. However, the difference between the spatial extent of the two buffers will depend on the street connectivity of the study area. In areas with higher street connectivity, the differences between the crow flies’ and street network buffers in terms of their spatial extent will be usually larger. Moreover, areas with high population density are usually areas where the street network connectivity is higher [26].

In the literature, there is not a standardized methodology to measure alcohol outlet density in urban areas. As discussed in the “Introduction” section, this might be the reason why different studies that related alcohol outlet density with patterns of consumption concluded different findings. These kinds of disparities have been highlighted in other environments related to urban health, as well. A systematic review about the tobacco environment discussed the association between tobacco consumption and its accessibility and availability approached using different methodologies [18]. This work found that the use of different methods affected the direction and magnitude of the association between exposure and outcomes. For example, most of the studies that used KDE found positive associations between tobacco outlet density and smoking prevalence. Also, those studies which estimated tobacco outlet densities as counts within crow flies’ buffers reported a higher number of positive associations with smoking prevalence than those which used counts within street network buffers [18]. Thus, future research should not only be focused on finding the best methodology, but in trying to unify which methodology should be used by all researchers according to the geographical and morphological characteristics of the area, in order to be able to compare studies carried in different geographical zones [27].

Our results enlight us that, depending on the characteristics of the study area, the results of the estimated alcohol outlet densities might vary depending on the type and size of the chosen buffer. This study was developed in the city of Madrid, which is characterized by a dense urban morphology (with a high percentage of the population concentrated in a limited space, living in tall-building apartments), higher degrees of land use diversification, and a generally irregular and dense street network pattern [28]. This type of urban morphology is typical amongst European and Mediterranean cities. Other settings, such as Anglo-Saxon countries, are characterized by a less densely urban morphology with a higher urban sprawl throughout regular street network patterns which leads to lower population densities (e.g., Melbourne in Australia presented a lower population density compared to Madrid, 2640.65 vs 5459.51 inhabitants per sq. km.) and a segregated land use distribution [29, 30]. Understanding and recognizing these particular geographies are crucial when examining which type of exposure measure would be the most appropriate to our analyses in our study setting.

Our findings can also be useful in order to propose future alcohol legislation at a national and international level. It has been proven that higher densities of alcohol outlets (measured with both, crow flies’, and street network buffers) around schools and residences predict higher alcohol consumption in adolescents [31, 32]. Thus, policies should focus on reducing alcohol outlet availability and accessibility by establishing minimum distances between schools and alcohol outlets, in order to decrease exposure and opportunities to buy alcohol in adolescents. For example, in Ireland, The Public Health (Alcohol) Act (2018) outlawed alcohol advertising within 200 m from schools [33], and in California, the minimum distance from schools for new licensed liquor stores has to be 600 ft (182.88 m) [34]. However, they do not specify if those buffers are measured using crow flies’ or street network distances. In this line, it may be interesting to use buffers in Madrid to establish the limits from which the alcohol outlets should not be present. Then the most pertinent type of measure to do so should be evaluated accounting with the geographical caracteristics of the study area such as the urban morphology (e.g., street connectivity, population density, and recreational venues density).

In the case of our study conducted in Madrid, future policies applying crow flies’ buffers to restrict alcohol outlet availability near schools might achieve better outcomes. Similar results have been evinced by other studies in tobacco control research and might be also extrapolated to other settings [22]. Moreover, these policies might be useful to tackle other public health issues, such as the health inequalities caused by the differences in alcohol outlet density found around schools depending on the SES of the area [21, 35].

Our study has some limitations. First, the study was restricted to an urban area with high street connectivity and population density. It would be interesting to do similar studies in rural areas and in urban areas where the land use distribution might be vastly different [29], especially since we found that the differences between methods vary according to these characteristics. Moreover, another limitation is that there is not a comparison between the association of drinking behaviors with the exposure measurements defined by different types of buffers. Future studies could dive into this question, using standard and consistent methods, and comparing if one method gives more positive associations with drinking patterns than the other.

This study suggests that characteristics and morphology of the study area, such as street network connectivity (accounted here as the density of street intersections) or urban compactness (approached here as population density), constitute important factors to consider when assessing the most suitable method to calculate exposure measures to approach alcohol availability. From a policy perspective, our results could anticipate that future interventions focused on crow flies’ buffers might lead to a higher percent of reductions in the alcohol outlet densities around the schools in Madrid. Similar results could be valid for other international settings. Finally, the methodological steps performed in this study (i.e., Bland-Altman, cartography and regression) might be replicated in future studies based on different international settings when analyzing the appropriate method to approach alcohol outlet exposure around schools.

## Supplementary Information

ESM 1: Figures S1-S2 (DOCX 132 kb)
