# Supplementary material for: How Can We Measure Alcohol Outlet Density Around Schools? A Comparison Between Two Buffer-Based Methods
Source: J Urban Health. 2023 Jun 23;100(3):627–37. doi: 10.1007/s11524-023-00740-z (PMC10323074; doi:10.1007/s11524-023-00740-z)
Supplement: Supplementary file 1 — Figures S1-S2 (DOCX 132 kb) [file 11524_2023_740_MOESM1_ESM.docx]

**SUPPLEMENTARY MATERIAL**


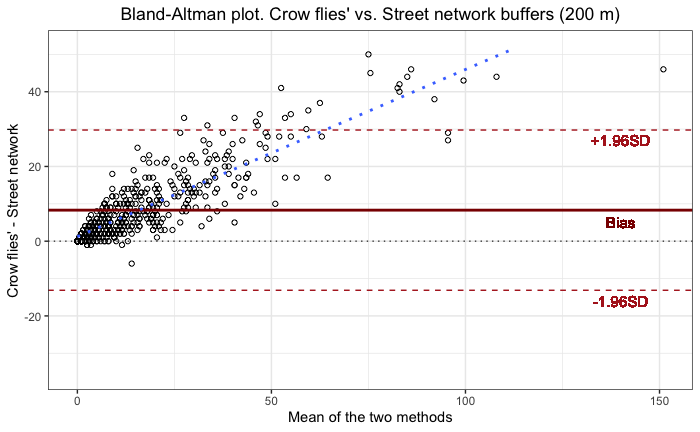


Supplemental Figure 1. Bland-Altman plot for the 200-meter buffers.


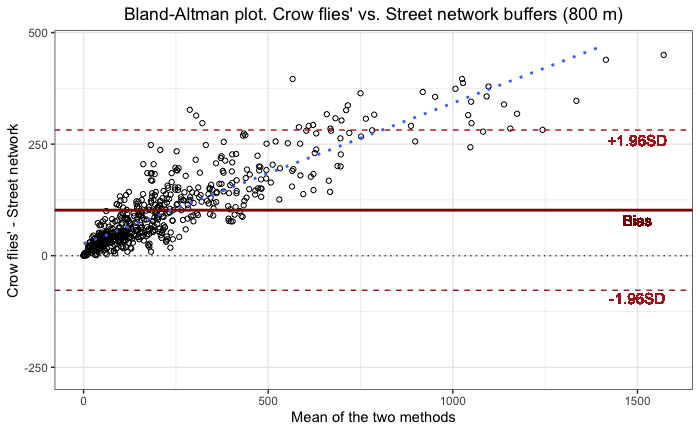


Supplemental Figure 2. Bland-Altman plot for the 800-meter buffers.
